# Size and competitive mating success in the yeast *Saccharomyces cerevisiae*

**DOI:** 10.1093/beheco/art117

**Published:** 2013-12-23

**Authors:** Carl Smith, Andrew Pomiankowski, Duncan Greig

**Affiliations:** ^a^The Galton Laboratory, Department of Genetics, Evolution, and Environment, University College London, Gower Street, London WC1E 6BT, UK,; ^b^CoMPLEX, University College London, Gower Street, London WC1E 6BT, UK, and; ^c^Max Planck Institute for Evolutionary Biology, August Thienemann Strasse 2, Plön 24306, Germany

**Keywords:** body size, cell size, mate choice, mating, *Saccharomyces cerevisiae*, sexual selection.

## Abstract

In unicellular organisms like yeast, mating with the right partner is critical to future fitness because each individual can only mate once. Because cell size is important for viability, mating with a partner of the right size could be a significant advantage. To investigate this idea, we manipulated the size of unmated yeast cells and showed that their viability depended on environmental conditions; large cells do better on rich medium and small cells do better on poor medium. We also found that the fitness of offspring is determined by the size of their parents. Finally, we demonstrated that when a focal cell of one mating type was placed with a large and a small cell of the opposite mating type, it was more likely to mate with the cell that was closer to the optimum size for growth in a given environment. This pattern was not generated by differences in passive mating efficiency of large and small cells across environments but by competitive mating behavior, mate preference, or both. We conclude that the most likely mechanism underlying this interesting behavior is that yeast cells compete for mates by producing pheromone signals advertising their viability, and cells with the opportunity to choose prefer to mate with stronger signalers because such matings produce more viable offspring.

## Lay Summary

Yeast cells that are too big or too small are more likely to remain virgins. Big yeast cells are fitter than small cells when food is plentiful, but smaller cells are fitter when food is scarce. When there is a choice of different size potential mates, the best size partner for the conditions is more likely to be chosen for sex, ensuring that the resulting offspring are of a fit size.

## INTRODUCTION

In most species, body size is fundamental to fitness. Classical work by Hermon T. Bumpus on sparrows knocked out by a winter storm showed how important body size was for survival (Bumpus 1899). Birds at the extremes of the size range—those that were either very large or very small—were more likely to survive than those of intermediate size. Selection can also explain why females are larger than males in most animals and dioecious plants (Fairbairn et al. 2007). Eggs are larger than sperm or pollen, so in many species, female fecundity is limited by the number of eggs or offspring that can be produced, selecting for large body size, but male reproductive success is typically limited by the number of eggs that can be fertilized, selecting for traits that increase mating success. The effect fecundity selection can have on body size can be spectacular; for example, some angler fish females can be half a million times heavier than males (Pietsch 2005).

Selection due to competition between males for access to females often favors larger males, opposite to the usual effect of selection for fecundity (Andersson 1994; Fairbairn et al. 2007). In most mammals and birds (except predatory and flightless birds), males are larger than females probably because larger males are better at fighting or competing for females (Gaulin and Sailer 1984). Females may get direct benefits from their mates and so choose larger males who can monopolize more beneficial resources than smaller males, for example, in bullfrogs, where more attractive larger males have better territories for egg laying (Howard 1978). Even when females do not benefit directly from larger males, they may prefer them because their offspring can benefit instead, for example, in crickets, where larger males produce fitter offspring (Simmons 1987). Size also can affect another component of selection that generated by female mate choice. Female size can affect both the mate sampling rules adopted by females and the strength of preference they exert in favor of particular male traits, especially where size is correlated with female condition (Cotton, Small, et al. 2006). For example, when female black field crickets are raised on a high-protein diet, they are heavier as adults and exert a stronger stabilizing preference on male calling frequency as well as a stronger directional preference for male calling rate (Hunt et al. 2005). In addition, large females respond more quickly to male calls that contained preferred stimuli (Hunt et al. 2005). A similar size dependence has been observed in 2 species of stalk-eyed flies, where females with large eye span show strong mate preference, whereas females with small eye span are much more indiscriminate with whom they mate (Hingle et al. 2001; Cotton, Rogers, et al. 2006). These patterns can significantly impact the strength of sexual selection on male traits involved in courtship display.

But despite evidence for strong selection, high heritable variance for body size remains within populations. This is shown by the rapid evolution of body size when artificial selection is applied, for example, in domesticated dogs (Wayne 1986) and laboratory mice (Wilson et al. 1971). It is also evident from rapid changes in size associated when populations become isolated on islands, for example, with large mammals shrinking and small mammals growing. This pattern, which is known as the “island rule” (Van Valen 1973), is thought to be the result of adaptation to reduced food abundance for large species and reduced competition from other small species (Lomolino 1985).

In contrast to the behavioral models applied to body size evolution in large organisms, most studies of size in microbes are biophysical (e.g., Chisholm 1992) or ecological (e.g., size-specific grazing or predation, Hansen 1997). An important limit on the size of unicellular organisms is given by the uptake of essential nutrients (ideas particularly developed for marine phytoplankton). This depends on the local nutrient concentration and diffusibility and limits an organism’s growth rate (Pasciak and Gavis 1974). As diffusion of nutrients to the cell surface scales with cell volume with an exponent of 1/3, but metabolic capacity scales with cell volume with an exponent of 3/4 (Mei et al. 2009), lower nutrient concentration favors smaller cells (Raven 1998). Bacterial cells dividing by binary fission in laboratory culture increase in size when nutrients are abundant (Henrici 1928), a phenomenon that has been well analyzed genetically (for review see, Chien et al. 2012). This pattern is observed in fission yeast (Fantes and Nurse 1977) and in budding yeast (Adams 1977), the subject of this study. Small, newly budded *Saccharomyces cerevisiae* cells grow until they achieve a critical size, when they can then produce new buds themselves (Johnston et al. 1977); this critical size is smaller when glucose is scarce (Adams 1977; Johnston et al. 1979). Because the critical size increases with the number of daughter buds already produced (Johnston et al. 1979), yeast populations always contain a distribution of cell sizes, even though the mean cell size responds plastically when conditions change (Alberghina et al. 1998). Genotype also affects yeast cell size: A systematic screen of the *S. cerevisiae* gene knockout library found a complex epistatic network containing about 500 genes with major effects on yeast cell size (Jorgensen et al. 2002). In addition, yeast isolated from natural sources vary in both cell size and fitness according to strain and genotype (Spor et al. 2008).

Although *Saccharomyces* cell size during asexual growth has been well studied as a model for eukaryotic cell size regulation, the fitness consequences of size variation in the sexual phase of the yeast life cycle have been rather neglected. When diploid cells are starved, they typically enter meiosis, each producing a tetrad of 4 haploid resistant spores. The ability of a diploid to undergo meiosis, like the ability to bud, depends on its age, size, environment, and genotype (Calvert and Dawes 1984; Zeyl et al. 2005; Gerke et al. 2006; Elrod et al. 2009). Spores remain dormant until they sense new nutrients, when they germinate into metabolically active haploid cells that can then attempt to mate. There are 2 mating types, *MAT*α and *MAT*a, which are monomorphic but produce attractive sex pheromones called α-factor and a-factor, respectively. Haploids express the receptor for the opposite mating pheromone but not for their own mating pheromone type, so can only detect the pheromone produced by the opposite mating type (Dohlman and Thorner 2001). A pair of courting cells respond to each other’s pheromones by increasing their own pheromone outputs, changing shape to touch each other, and finally fusing together to form a diploid zygote. Most mating is believed to be selfing, occurring between haploids from the same meiotic tetrad (Greig and Leu 2009), but recent evidence suggests that mating between haploids from different tetrads is common in wild (Murphy and Zeyl 2010) and dispersing (Reuter et al. 2007) yeast. Haploid cells that fail to mate can instead divide asexually by haploid mitosis and have an opportunity to mate the following cell cycle, either with a cell from another lineage or after switching mating type, with their own mitotic progeny (Greig and Leu 2009).

When 2 yeast cells mate, their cells walls adhere and then break down, allowing their plasma membranes to touch and form a fusion pore, which expands to allow cytoplasmic mixing and karyogamy (Chen et al. 2007). The plasma membranes remain contiguous throughout, and no cytoplasmic material is gained or lost; unlike many other eukaryotes, yeast zygotes receive all mitochondria from both parents (Birky 1975). Therefore, the initial volume of a yeast zygote is simply the sum of the volumes of the 2 haploids that produced it. Because size is fundamental to cell division in budding yeast, there should be benefits to mating with a partner of the appropriate size in order to create a zygote whose initial size is close to the critical size for the local environment. Here, we simulate a natural scenario in which haploid spores of different sizes disperse to a new food resource, where they germinate and mate. We first verify that haploid spore size affects the viability of the resulting diploids in high- or low-glucose environments, and we then determine whether spores of a more viable size are more likely to mate. We analyze 1) whether spore size merely affects passive mating efficiency or 2) whether more complex behavior, such as mate competition between different sized cells or preference for different size mates, can be inferred.

## MATERIALS AND METHODS

### Strain design

We wanted to isolate the effect of phenotypic variation in cell size from the effect of genetic variation, so all experiments were carried out using a single, isogenic strain, Y55 (McCusker and Haber 1988). To determine which cell mated with which, auxotrophic genetic markers were used to create 2 distinguishable homozygous diploid parents, Parent A (*MAT*a*/MAT*α *ho/ho ura3/ura3 arg1/arg1*) and Parent B (*MAT*a/*MAT*α *ho/ho lys2/lys2 his4/his4*). These markers allowed the parent diploids, their haploid gametes, and new diploid zygotes resulting from their matings to be identified. All cells used were genetically identical apart from these markers and any new mutations that may have occurred spontaneously during the course of the experiment.

### Measuring the effect of potassium acetate concentration on spore size

To produce the different size spores used in these experiments, Parent A and Parent B were first spread in patches onto the surface of standard yeast medium (YEPD: 2% glucose, 2% peptone, 1% yeast extract, and 2.5% agar) and incubated at 30 °C for 24h before transferring the diploid cells by replica plating to sporulation medium consisting of 2% agar supplemented with either 2% or 0.01% potassium acetate. These plates were incubated at 25 °C for at least 7 days to allow the diploid cells to undergo meiosis and produce haploid spores. In subsequent parts of the paper, we will refer to spores produced on 2% potassium acetate as “large” and those produced on 0.01% potassium acetate as “small,” while recognizing that spores from these 2 media may also differ in ways other than size.

The effect of the 2 types of sporulation media on spore size was determined by measuring the resulting spores microscopically, taking precautions to prevent experimenter bias. Spores were scraped from the surface of the sporulation media, suspended in 0.025% zymolyase, and incubated at 25 °C for 4h to digest the outer asci but leave the spores associated in tetrads. A microscope was used to photograph at least 20 tetrads from each of the 4 samples (small or large spores from Parent A or B). A 50-µm hemocytometer scale was also photographed to calibrate the size of the images.

The 94 resulting images were randomized and renamed, so that the experimenter (D.G.) did not know which image came from which slide. Each image was opened with Image J (Schneider et al. 2012), and the diameter of a single spore from each image was measured. Three images were discarded because they were out of focus. The 50 µm scale was also measured and used to convert the pixel measurements into micrometer. Spore diameters were divided by 2 to yield measurements of spore radii (*r*), which were then converted into spore volumes (4/3 π *r* ^3^). These 91 values were then decoded to assign each back to one of the 4 samples (Supplementary Table S1). Data were analyzed by Generalized Linear Model (GLM) in JMP 9.0 (SAS Institute Inc., Cary, NC).

### Measuring the effect of spore size on haploid spore budding time

Large and small spores of Parent A were produced and digested with zymolyase as described above. We used a tetrad-dissecting micromanipulator to place individual spores on the surface of agar plates. Only one spore was used from any tetrad because we only used one spore from each tetrad in the mating assays (see Measuring the effect of spore size on mating, below). Two types of agar were used, rich medium, as described above, and poor medium, which is identical except that it contains 10-fold less glucose (i.e., 0.2% glucose, 2% peptone, 1% yeast extract, and 2.5% agar). The plates were incubated at 30 °C for 4h (for rich medium) or 6h (for poor media), and then the spores were observed every 10min until the first bud appeared. Twenty measurements were made in each of the 4 combinations of size and medium (Supplementary Table S2). Data were analyzed by GLM in JMP 9.0 (SAS Institute Inc.).

### Measuring the effect of spore size on diploid zygote budding time

We made zygotes from either 2 large spores or 2 small spores and measured how long it took them to produce their first offspring. Small or large spores were cultured and then treated with zymolyase, as described above. We used the tetrad-dissecting micromanipulator to pair large spores from Parent A with large spores from Parent B and small spores from Parent A with small spores from Parent B. Only one spore was used from any tetrad because we only used one spore from each tetrad in the mating assays (see Measuring the effect of spore size on mating, below). Pairs of spores were placed on the surface of an agar plate so that they touched, incubated at 30 °C for 4h, and then observed every 10min until either they fused together to form zygotes or budded without mating. We continued to observe new zygotes to measure the time it took between zygote formation and the production of a bud that could be removed by micromanipulation. The newly budded daughter cells were moved to a new part of the plate and allowed to form colonies, which were then tested by replica plating to minimal medium (2% glucose, 0.67% yeast nitrogen base without amino acids, and 2% agar) to verify that they were prototrophic and therefore came from diploid zygotes. Seventeen large zygotes and 11 small zygotes were measured on rich medium; 5 large zygotes and 5 small zygotes were measured on poor medium (Supplementary Table S3). Data were analyzed by GLM in JMP 9.0 (SAS Institute Inc.).

### Measuring the effect of spore size on mating

**Figure 1:**
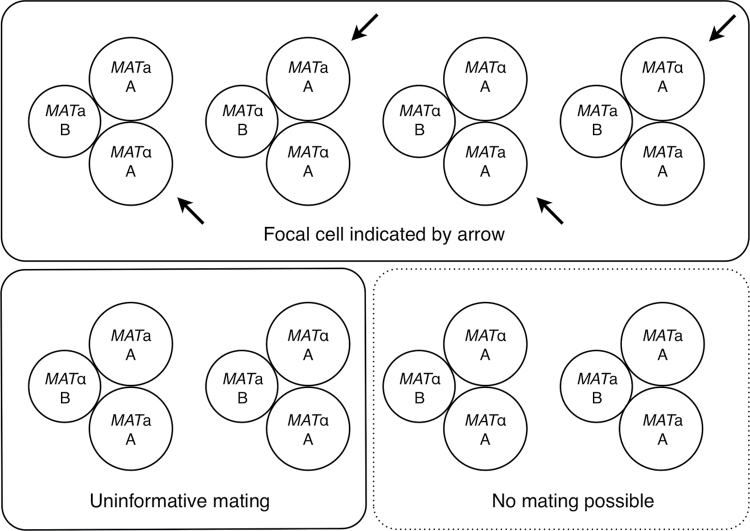
The 8 possible mating type and size combinations in each mating trial. In this example, 2 large spores from Parent A are placed with 1 small Parent B spore. The top 4 out of the 8 possible combinations create the interesting situation of a large focal cell (indicated by the arrow) that can mate with either a large or a small partner of the opposite mating type. The left-hand bottom 2 combinations also allow mating but only between large and small cells; there is no opportunity for size-specific mating. The right-hand bottom 2 combinations do not allow any mating because all spores are the same mating type. Thus, if mates were chosen randomly with respect to spore size, two-thirds of all matings are expected to be between small and large spores.

We conducted mating trials to determine how differences in the sizes of 2 competing spores of the same mating type affected their likelihood of mating with a single focal cell of the opposite mating type, on both rich and poor medium. Each trial comprised 3 spores: 2 of the same size class from one parent (one of which was the focal cell) and 1 of the other size class from the other parent (Figure 1). The different genetic markers carried by the parents and inherited by their haploid gametes allowed us to determine which size partner had mated with the focal cell. Tetrad asci were treated with zymolyase as above, and the 3 spores in each trial were placed on the surface of the medium using a micromanipulator. Each trial was observed until either a zygote formed or an unmated haploid budded. When a zygote formed, the third unmated haploid was removed to a different part of the plate, leaving the zygote to grow as a pure diploid colony whose genotype could be tested by replica plating to minimal medium as above. If the removed haploid failed to produce a colony, it was deemed to be dead, and the trial was excluded from the results.

Tetrads contain 2 spores of each mating type (*MAT*a and *MAT*α), which cannot be distinguished until the spores germinate and express the pheromone specific to each type. To ensure that mating types were sampled randomly, only one spore was sampled from any tetrad. There are, therefore, 8 equally probable combinations of size and mating type possible in each trial (Figure 1). Two combinations do not allow any mating because all spores are the same mating type (either all *MAT*a or all *MAT*α). Another 2 possible combinations are not informative because the focal cell does not have the possibility of mating with either a large or a small partner. But the remaining 4 combinations allow 2 cells of different sizes to compete in order to mate with a focal cell and for the focal cell to choose between 2 potential mates of different sizes. To control for any effect of the parent’s genetic markers on mating behavior, the trials were done on rich medium with all 4 combinations of size and parent: 2 large Parent A spores with 1 small Parent B spore (large Parent A spore as focal cell), 2 large Parent B spores with 1 small Parent A spore (large Parent B spore as focal cell), 2 small Parent A spores with 1 large Parent B spore (small Parent A spore as focal cell), and 2 small Parent B spores with 1 large Parent A spore (small Parent B spore as focal cell). After it was established that the genetic markers had no detectable effect on mating, the trials were repeated on poor medium but without reversing the markers: 2 large Parent A spores with 1 small Parent B spore (large Parent A spore as focal cell) and 2 small Parent A spores with 1 large Parent B spore (small Parent A spore as focal cell). A total of 709 trials were done (Table 1).

**Table 1:** Mating trial results

Focal cell size	Medium	Focal cell parent	Total trials	Total matings	Matings with large spore
Large	Rich	A	120	61	30
Large	Rich	B	119	56	28
Small	Rich	A	117	56	47
Small	Rich	B	120	59	50
Large	Poor	A	120	62	10
Small	Poor	A	113	65	30

Given this design, if mating is random with respect to size, two-thirds of all matings were expected to be between small and large spores (see Figure 1). For each of the 6 combinations of size and parent above, we tested whether the number of observed matings between small and large spores (determined by the complementation of their genetic markers) differed significantly from this expectation using a χ^2^ goodness of fit test.

For illustration, we calculated a measure of mating advantage of large spores from the proportion of matings with large spores from the total number of mated trials. In trials with large spores as the focal cell, one-third of the matings would have resulted from uninformative trials where there was no size competition or choice and mating could only occur between a large and a small spore (Figure 1). We corrected for this, so the mating advantage of large spores (*P*_*L*_) is given by:

$$P_{L}\text{=}\frac{\text{3}N_{LL}}{\text{2}\left(N_{LL}\text{+}N_{LS}\right)},$$

where *N*_LL_ is the number of matings between 2 large spores and *N*_LS_ is the number of matings between large and small spores (first subscript letter indicates the focal cell). The equivalent calculation of the mating advantage of large spores for trials in which the small spore was the focal cell is (again one-third of the matings are uninformative):

$$P_{L}\text{=1}-\frac{\text{3}N_{SS}}{\text{2}\left(N_{SS}\text{+}N_{SL}\right)}$$

Both formulae give an expected mating advantage of large spores between 0 and 1 (stochastic variation in the proportion of each type of mating could give values that exceed these bounds). A value of greater than 0.5 indicates that large spores have a mating advantage over small spores, a value of smaller than 0.5 indicates a mating advantage for small spores over large spores.

## RESULTS

Raw data are provided for all experiments: spore sizes in Supplementary Table S1, haploid spore budding times in Supplementary Table S2, diploid zygote budding times in Supplementary Table S3, and mate choices in Table 1.

### The effect of potassium acetate concentration on spore size

The concentration of potassium acetate in the sporulation medium had a strong and significant effect on the spore size (*F*_1, 87_ = 59.86, *P* < 0.0001); spores from 2% potassium acetate were larger (46.29 µm^3^, standard deviation [SD] = 9.22 µm^3^, *n* = 45) than those from 0.001% potassium acetate (32.64 µm^3^, SD = 7.88 µm^3^, *n* = 46). After taking medium into account in a GLM, there was a significant effect of parent (*F*_1, 87_ = 4.55, *P* = 0.036), although there was no evidence that the 2 parents behaved differently on the 2% and 0.001% media (interaction term, *F*_1, 87_ = 1.64, *P* = 0.20).

### The effect of spore size on haploid spore budding time

**Figure 2:**
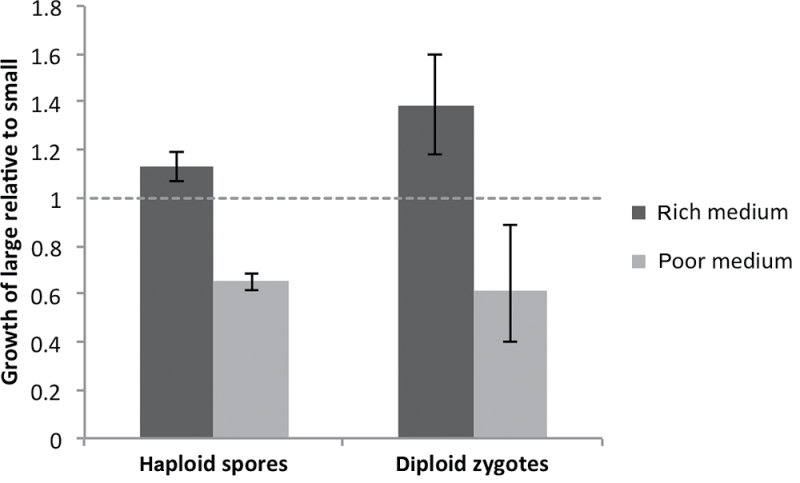
Initial growth rate of large cells relative to small cells on different media, for haploid spores and for diploid zygotes. This measures the asexual fitness of large cells relative to small cells for the first cell cycle after germination (for spores) or after mating (for zygotes). This measure of viability is calculated as the mean time taken for small cells to produce a bud divided by the mean time taken for large cells to produce a bud. The dotted line at 1.0 indicates equal viability; values above the line indicate that large cells grow faster than small cells. Error bars show SDs.

The time taken between placing a spore on the surface of an agar plate and the appearance of the first mitotic bud was measured for large and small spores on rich and poor media. Using a GLM, we showed that both spore size (*F*_1, 76_ = 5534.06, *P* < 0.0001) and medium type (*F*_1,76_ = 1195.33, *P* < 0.0001) had significant effects on spore budding time, and these factors interacted strongly (*F*_1, 76_ = 2424.39, *P* < 0.0001) because on rich medium, large spores divided faster than small spores (*F*_1, 38_ = 193.83, *P* < 0.0001), but on poor medium, small spores divided faster than large spores (*F*_1, 38_ = 2430.124, *P* < 0.0001). These results are summarized in Figure 2.

### The effect of spore size on diploid zygote budding time

To assess the effect of haploid spore size on diploid viability, the average time between zygote formation and completion of the first diploid cell division was measured. Both the size of the spores that mated to create the zygotes (*F*_1, 34_ = 31.31, *P* < 0.0001) and the medium type (*F*_1, 34_ = 459.2, *P* < 0.0001) had significant effects on the time it took for zygotes to divide. There was also a strong interaction (*F*_1, 34_ = 55.55, *P* < 0.0001), because on rich medium, zygotes made from large spores divided faster than zygotes made from small spores (*F*_1, 26_ = 77.66, *P* < 0.0001), but on poor medium, the reverse was true and zygotes made from small spores were favored (*F*_1, 8_ = 14.21, *P* = 0.0055). The results are summarized in Figure 2.

### The effect of spore size on mating

**Figure 3:**
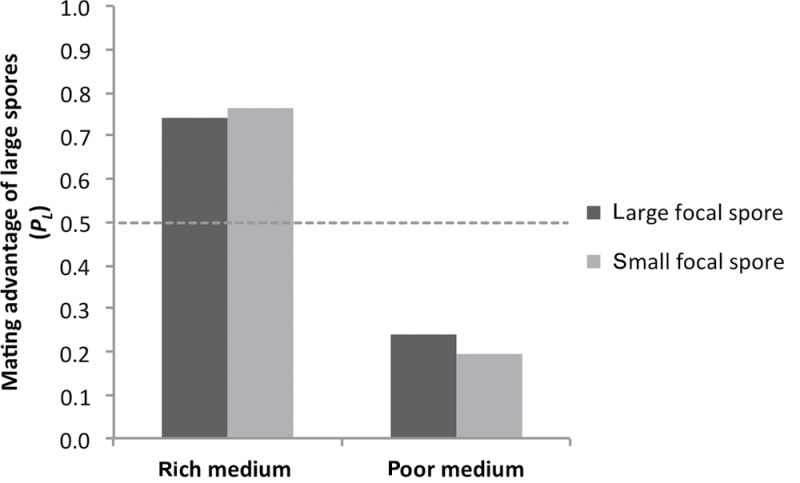
Mating advantage of large spores when the focal spore can mate with either a large or a small haploid spore in a mating trial. The mating advantage was determined for when focal spores are large (dark grey bars) or small (light grey bars), on rich or poor medium. If mating was random with respect to size, large and small spores would be equally likely to mate, and the mating advantage of large spores would be 0.5 (neutral, indicated by the dashed line). Values above 0.5 indicate that large cells have a mating advantage and values below 0.5 mean that smaller cells have a mating advantage.

Different size focal cells (large or small), on different media (rich or poor), were offered 2 potential partners of different sizes (large or small). As described in Materials and Methods, the null probability of mating with a large partner depends on the size of the focal cell (large focal cell: *P* = 1/3, small focal cell: *P* = 2/3), and this was taken into account in the following tests. The pattern of mating across these 4 categories (2 sizes × 2 media) was nonrandom (χ^2^ = 50.6, df = 7, *P* < 0.001). Within each size and media class, there was nonrandom mating in favor of the mate who would produce fitter sized offspring (Figure 3): On rich medium, both the large (χ^2^ = 13.9, df = 1, *P* < 0.001) and small focal cells (χ^2^ = 16.2, df = 1, *P* < 0.001) were more likely to mate with large partners; and on poor medium, both the large (χ^2^ = 8.3, df = 1, *P* = 0.004) and small focal cells (χ^2^ = 12.4, df = 1, *P* < 0.001) were more likely to mate with small partners.

In the experiments on rich medium, both parents, which carry different genetic markers, were used to make focal cells, which could potentially have been a confounding variable. But we found that the parent genetic markers had no effect on the frequency of mating with large cells when the focal cell was large (2×2 contingency table, χ^2^ = 0.0078, df = 1, *P* = 0.93) or when the focal cell was small (χ^2^ = 0.0014, df = 1, *P* = 0.90). So, genetic differences due to auxotrophic markers did not underlie the pattern of nonrandom mating.

To see whether our results could be explained by differences in simple mating efficiency between large and small spores, we tested whether the size of the focal cell affected how many trials resulted in mating. However, there was no difference in the number of matings with large or small focal cells either in general (2×2 contingency table, χ^2^ = 0.17, df = 1, *P* = 0.68), on rich medium (χ^2^ = 0.0088, df = 1, *P* = 0.93) or on poor medium (χ^2^ = 0.80, df = 1, *P* = 0.36). Thus, the bias toward matings with optimally sized cells is due to either a competitive mating advantage of more viable cells or a preference for more viable partners, or both.

## DISCUSSION

In *S. cerevisiae*, we expect that the size of mating cells will affect the immediate fitness of their offspring. Thus, natural selection should favor mechanisms that increase matings between optimally sized cells when a choice of different size partners is available. We further expect that such mechanism should not lead to a reduction in mating ability when there is no variation in mate size. We investigated what happens when spores of different sizes disperse to new food resources, germinate, and mate. We found that there is indeed a bias toward matings with optimal sized partners, and that this is not due to cell size affecting passive mating efficiency. Rather, we conclude that the pattern of mating was caused by either active competition between different size cells of the same mating type for access to partners of the other type (analogous to sexual selection by male–male competition) or active mate choice for partners of the right size (analogous to sexual selection by female choice), or a combination of both.

### The benefits to zygotes of being the right size

The initial competitive advantage of being a diploid zygote of the right size is considerable. On rich medium, we found that zygotes derived from large spores reproduced 38% more quickly than zygotes with small parents, and on poor medium, zygotes with small parents were 63% quicker to reproduce (Figure 2 and Supplementary Table S3). Even if reproduction proceeds at the same rate after the initial division (as expected if newly budded cells grow at equal rates irrespective of the size of their genetically identical parents), zygotes that are initially closer to the optimal size should ultimately produce more offspring in proportion to the size of these “head-starts.” We manipulated spore size by changing the concentration of a carbon source, potassium acetate, in the sporulation environment. Thus, we cannot be certain that size was the only phenotypic difference between the large and small spores or, therefore, that volume was the only benefit inherited by their zygotes. It is reasonable to suppose that the nutrient level of the sporulation environment might change other phenotypic traits, such as the thickness of the spore wall or the concentration of stored nutrients, and these might cause us to underestimate or overestimate, respectively, the contribution of size to zygote viability.

In our study, in order to isolate the effect of cell size from genetic factors, we used isogenic parents whose initial viability was determined only by the sporulation environment. In nature, though, we would expect cell size to be determined also by genetic differences and gene-by-environment interactions (Spor et al. 2008). This means that progeny of a particular mating could inherit a genetic advantage affecting all subsequent mitotic divisions, compounding the relative benefits of mating with the right size partner. Thus the advantage of mating with more viable sized cells could have both the direct (phenotypic) benefits we have demonstrated here, as well as indirect (genetic) fitness benefits that extend throughout the life cycle.

### What mechanism underlies the mating advantage of fitter size spores?

We found that large and small spores differed in the time it took for them to produce a daughter cell by haploid mitosis. If there is a similar difference in the time it takes for large and small spores to become ready to mate, this might provide a simple mechanism determining which cells mate. Experimental evolution has shown that differences in mating dynamics in initially isogenic populations can evolve rapidly, leading to assortative mating (Leu and Murray 2006). In addition, laboratory tests show that mating between *S. cerevisiae* and the closely related species *Saccharomyces paradoxus* is reduced because *S. paradoxus* spores germinate more slowly and are not ready to mate at the same time as *S. cerevisiae* (Murphy et al. 2006; Maclean and Greig 2008; Murphy and Zeyl 2012). Consistent with the hypothesis that optimally sized spores become ready to mate sooner, we found a greater frequency of assortative matings between optimally sized cells on both media. Large focal cells were more likely to mate with large cells on rich medium and small focal cells were more likely to mate with small cells on poor medium.

But simple assortative mating is not sufficient to explain our results, because when the less viable size was the focal cell, it also tended to mate with the more viable sized partner rather than the less viable size. So, small focal cells on rich medium were more likely to mate with large cells, whereas large focal cells on poor medium were more likely to mate with small cells. This behavior cannot be due simply to greater mating availability or mating ability of more viable cells, because less viable focal cells mate as efficiently as more viable focal cells, and the mating advantage of more viable sized cells is not affected by whether the focal cell is large or small (Figure 3).

Differences in the amount of sex pheromone produced by different sized spores could explain the mating patterns we observe. Yeast haploids use the pheromone signaling system to court and choose partners, and cells that produce more pheromone are more competitive at courting and more likely to be chosen as partners (Jackson and Hartwell 1990). A more viable spore that germinates more quickly might begin to produce pheromone earlier, allowing more pheromone to build up around it and making it more attractive than a spore that begins producing pheromone later. Another explanation is that more viable haploids can produce pheromone at a higher rate than less viable haploids (Pagel 1993; Tazzyman et al. 2012). Recent experiments support this theory, showing that the metabolic cost of producing sex pheromones is lower for more viable strains, allowing better quality individuals to signal more strongly, making them more attractive (Smith and Greig 2010). As large spores are better on rich medium and small spores are better on poor medium, they are both predicted to produce higher levels of pheromone, either by beginning pheromone production earlier or by producing it at a higher rate, or both. Given that focal cells are more likely to mate with the partner that produces the most pheromone (whether this is the longer or the stronger signaler, or both), this will result in assortative mating when the focal cell is the more viable size and disassortative mating when the focal cell is not.

Throughout this paper, we have considered the mating behavior of yeast in 2 contexts drawing from the standard model of sexual selection (i.e., male–male competition and female choice, Andersson 1994). We can interpret the behavior as competition between potential partners that are signaling for the focal cell’s attention, in which case the mating advantage of more viable cells (Figure 3) could be considered as measure of competitive mating ability, such as “attractiveness.” We cannot discount the formal possibility that the mating advantage is due to a direct interaction with competitors, perhaps by blocking them physically or interfering with their pheromone signals. Although such a mechanism would be closely analogous to male–male competition, we think it unlikely because cells do not express the receptor for their own pheromone type, so we do not expect them to be able to assess the presence of their competitors (Dohlman and Thorner 2001). Our favored interpretation is that of a focal cell choosing between potential partners, which are signaling for the focal cell’s attention, in which case the mating advantage of more viable cells (Figure 3) could be considered a measure of “mate preference.” In order to detect this behavior, our experimental trials were set up, so that 2 cells would be competing to mate with a third cell, which could choose between them. In natural situations, whenever there are more than 2 haploids present, there is the possibility that both mate competition and mate choice will affect mating outcomes, and sexual selection should act to optimize both competitive and choosy mating strategies. Overall, there are equal numbers of both mating types, so on average, we expect mutual mate choice to produce assortative mating between the fittest available individuals (Tazzyman et al. 2012).

## CONCLUSION

For a single-celled organism like yeast, mating is an absolute commitment that fuses 2 individuals into one; it is an irreversible union. We, therefore, expect that a yeast cell should choose the best possible partner. Here, we have looked at an important determinant of mate quality, cell size, and found evidence that yeast cells of different sizes tend to mate in ways that optimize the size and hence fitness of their resulting offspring.

## SUPPLEMENTARY MATERIAL

Supplementary material can be found at http://www.beheco.oxfordjournals.org/

## FUNDING

C.S. was supported by a Biotechnology and Biological Sciences Research Council studentship, A.P. was supported by grants from the Natural Environment Research Council (NE/G00563X/1) and the Engineering and Physical Sciences Research Council (EP/F500351/1 and EP/I017909/1), and D.G. by the Royal Society and the Max Planck Society.

## Supplementary Material

Supplementary Data
