# Supplementary material for: Size and competitive mating success in the yeast Saccharomyces cerevisiae
Source: Behav Ecol. 2013 Dec 23;25(2):320–7. doi: 10.1093/beheco/art117 (PMC3945744; doi:10.1093/beheco/art117)
Supplement: Supplementary Data [file supp_25_2_320__index.html]

Size and competitive mating success in the yeast Saccharomyces cerevisiae — Size and competitive mating success in the yeast Saccharomyces cerevisiae — Supplementary Data 

# Size and competitive mating success in the yeast *Saccharomyces cerevisiae*

## Supplementary Data

Data files

**Files in this Data Supplement:**

- Supplementary Data - Supplementary Data
