# Supplementary material for: Size and competitive mating success in the yeast Saccharomyces cerevisiae
Source: Behav Ecol. 2013 Dec 23;25(2):320–7. doi: 10.1093/beheco/art117 (PMC3945744; doi:10.1093/beheco/art117)
Supplement: Supplementary Data [file supp_art117_Supplementary_Material.doc]

Table S1.

Spore size measurements in μm3 for the two Parents (A and B) from the two sporulation media (2% and 0.01% potassium acetate).

| 2%, Parent A | 2%, Parent B | 0.01%, Parent A | 0.01%, Parent B |
| --- | --- | --- | --- |
| 36.5832978 | 41.84935155 | 19.84550957 | 29.32726645 |
| 34.36295323 | 44.50470401 | 29.55944794 | 20.29658703 |
| 26.6532556 | 42.69005754 | 33.08653526 | 36.84336062 |
| 35.86456464 | 43.0592098 | 32.31477687 | 28.68716302 |
| 47.91493978 | 33.70878391 | 34.36295323 | 29.11285187 |
| 49.01472521 | 33.23233354 | 32.54598998 | 37.54941731 |
| 57.40585057 | 51.6647181 | 23.23480994 | 32.04664159 |
| 59.33701232 | 56.62860725 | 48.37607378 | 29.72825312 |
| 46.34575396 | 46.39958446 | 48.46364682 | 34.01151346 |
| 65.80292673 | 61.07493729 | 48.49584304 | 32.54598998 |
| 57.98838834 | 34.22662628 | 32.85414605 | 30.71499666 |
| 59.65480795 | 43.47205281 | 36.5832978 | 27.34946028 |
| 57.40585057 | 49.78995279 | 47.3246114 | 24.59019538 |
| 46.00982446 | 56.31573179 | 31.70177102 | 28.68716302 |
| 57.42987785 | 51.57519297 | 41.17035189 | 21.25835106 |
| 47.57499321 | 42.43749312 | 31.23512907 | 24.76580639 |
| 52.40914071 | 49.42306372 | 33.94236048 | 40.68146376 |
| 38.94054435 | 42.60630541 | 49.73353049 | 27.22265068 |
| 44.98795511 | 41.84935155 | 44.15252793 | 31.21645256 |
| 39.27845501 | 39.67932146 | 31.28318858 | 20.65908952 |
| 47.25951566 | 49.43393536 | 32.57479741 | 29.5787443 |
| 27.18614397 |  | 30.08340191 | 42.69005754 |
| 38.32863426 |  | 26.50946709 | 22.48069922 |
| 53.77932451 |  |  |  |

Table S2

Haploid spore initial budding times in minutes for different size spores on different media.

| large spores on rich medium | small spores on rich medium | large spores on poor medium | small spore on poor medium |
| --- | --- | --- | --- |
| 300 | 330 | 630 | 400 |
| 300 | 345 | 600 | 420 |
| 300 | 345 | 615 | 390 |
| 275 | 330 | 600 | 400 |
| 300 | 330 | 600 | 390 |
| 315 | 345 | 600 | 400 |
| 300 | 330 | 630 | 390 |
| 300 | 345 | 630 | 390 |
| 300 | 345 | 630 | 420 |
| 300 | 345 | 645 | 400 |
| 300 | 330 | 615 | 390 |
| 300 | 330 | 615 | 400 |
| 275 | 330 | 600 | 420 |
| 300 | 345 | 645 | 400 |
| 300 | 330 | 630 | 420 |
| 300 | 330 | 630 | 420 |
| 300 | 330 | 600 | 390 |
| 300 | 345 | 600 | 400 |
| 300 | 345 | 615 | 390 |
| 315 | 330 | 615 | 400 |

Table S3

Diploid zygote initial budding times in minutes for zygotes formed from large or small spores on rich or poor media.

| large zygotes on rich medium | small zygotes on rich medium | large zygotes on poor medium | small zygotes on poor medium |
| --- | --- | --- | --- |
| 79 | 100 | 470 | 350 |
| 79 | 80 | 537 | 251 |
| 83 | 125 | 667 | 249 |
| 83 | 110 | 582 | 390 |
| 83 | 110 | 598 | 510 |
| 83 | 126 |  |  |
| 83 | 126 |  |  |
| 80 | 110 |  |  |
| 80 | 110 |  |  |
| 80 | 126 |  |  |
| 79 | 126 |  |  |
| 79 |  |  |  |
| 85 |  |  |  |
| 85 |  |  |  |
| 85 |  |  |  |
| 85 |  |  |  |
| 85 |  |  |  |
